# Supplementary material for: Modeling glioblastoma heterogeneity as a dynamic network of cell states
Source: Mol Syst Biol. 2021 Sep 16;17(9):e10105. doi: 10.15252/msb.202010105 (PMC8444284; doi:10.15252/msb.202010105)
Supplement: Supplementary file 6 — Source Data for Figure 5 [file MSB-17-e10105-s004.zip › Figure5A_sourcedata/GSEA_3017/nosigdb_stateE.GseaPreranked.1622795031093/SEURAT_CELLCYCLE.html]

Details for gene set SEURAT\_CELLCYCLE[GSEA]

|  || Dataset | state63017 |
| Phenotype | NoPhenotypeAvailable |
| Upregulated in class | na\_neg |
| GeneSet | SEURAT\_CELLCYCLE |
| Enrichment Score (ES) | -0.68103194 |
| Normalized Enrichment Score (NES) | -3.4536037 |
| Nominal p-value | 0.0 |
| FDR q-value | 0.0 |
| FWER p-Value | 0.0 |
Table: GSEA Results Summary

  

Fig 1: Enrichment plot: SEURAT\_CELLCYCLE      
 Profile of the Running ES Score & Positions of GeneSet Members on the Rank Ordered List

  

| PROBE | GENE SYMBOL | GENE\_TITLE | RANK IN GENE LIST | RANK METRIC SCORE | RUNNING ES | CORE ENRICHMENT || 1 | MCM5 |  |  | 337 | -0.265 | -0.3848 | No |
| 2 | SLBP |  |  | 386 | -0.277 | -0.4353 | No |
| 3 | NASP |  |  | 410 | -0.285 | -0.4567 | No |
| 4 | CTCF |  |  | 443 | -0.297 | -0.4883 | No |
| 5 | MCM4 |  |  | 457 | -0.301 | -0.4979 | No |
| 6 | UBR7 |  |  | 459 | -0.302 | -0.4936 | No |
| 7 | RFC2 |  |  | 498 | -0.313 | -0.5319 | No |
| 8 | PRIM1 |  |  | 539 | -0.328 | -0.5722 | No |
| 9 | MSH2 |  |  | 548 | -0.332 | -0.5754 | No |
| 10 | CDC25C |  |  | 549 | -0.332 | -0.5694 | No |
| 11 | RAD51 |  |  | 553 | -0.335 | -0.5668 | No |
| 12 | DSCC1 |  |  | 587 | -0.352 | -0.5986 | No |
| 13 | G2E3 |  |  | 624 | -0.371 | -0.6335 | No |
| 14 | WDR76 |  |  | 626 | -0.373 | -0.6280 | No |
| 15 | NEK2 |  |  | 628 | -0.374 | -0.6223 | No |
| 16 | GAS2L3 |  |  | 647 | -0.388 | -0.6361 | No |
| 17 | GINS2 |  |  | 658 | -0.399 | -0.6405 | No |
| 18 | RPA2 |  |  | 688 | -0.424 | -0.6663 | No |
| 19 | CKAP5 |  |  | 694 | -0.426 | -0.6644 | No |
| 20 | RANGAP1 |  |  | 698 | -0.429 | -0.6601 | No |
| 21 | POLD3 |  |  | 710 | -0.437 | -0.6650 | No |
| 22 | TTK |  |  | 724 | -0.454 | -0.6718 | No |
| 23 | POLA1 |  |  | 733 | -0.460 | -0.6727 | Yes |
| 24 | BRIP1 |  |  | 734 | -0.461 | -0.6644 | Yes |
| 25 | LBR |  |  | 739 | -0.463 | -0.6606 | Yes |
| 26 | E2F8 |  |  | 741 | -0.467 | -0.6533 | Yes |
| 27 | CDCA3 |  |  | 759 | -0.497 | -0.6640 | Yes |
| 28 | GMNN |  |  | 766 | -0.510 | -0.6617 | Yes |
| 29 | CDC45 |  |  | 773 | -0.521 | -0.6593 | Yes |
| 30 | CENPA |  |  | 782 | -0.538 | -0.6588 | Yes |
| 31 | NCAPD2 |  |  | 784 | -0.540 | -0.6502 | Yes |
| 32 | BLM |  |  | 786 | -0.543 | -0.6415 | Yes |
| 33 | CDC20 |  |  | 793 | -0.562 | -0.6383 | Yes |
| 34 | TUBB4B |  |  | 804 | -0.573 | -0.6395 | Yes |
| 35 | USP1 |  |  | 806 | -0.575 | -0.6302 | Yes |
| 36 | CDC6 |  |  | 807 | -0.584 | -0.6197 | Yes |
| 37 | CDCA8 |  |  | 808 | -0.585 | -0.6091 | Yes |
| 38 | CDCA2 |  |  | 816 | -0.597 | -0.6064 | Yes |
| 39 | AURKA |  |  | 824 | -0.623 | -0.6032 | Yes |
| 40 | CKAP2L |  |  | 826 | -0.625 | -0.5931 | Yes |
| 41 | AURKB |  |  | 829 | -0.634 | -0.5840 | Yes |
| 42 | HELLS |  |  | 830 | -0.635 | -0.5725 | Yes |
| 43 | DTL |  |  | 831 | -0.637 | -0.5610 | Yes |
| 44 | PCNA |  |  | 832 | -0.640 | -0.5494 | Yes |
| 45 | BUB1 |  |  | 837 | -0.653 | -0.5422 | Yes |
| 46 | ANP32E |  |  | 841 | -0.676 | -0.5334 | Yes |
| 47 | BIRC5 |  |  | 843 | -0.679 | -0.5223 | Yes |
| 48 | ECT2 |  |  | 854 | -0.717 | -0.5209 | Yes |
| 49 | KIF2C |  |  | 855 | -0.721 | -0.5079 | Yes |
| 50 | NUF2 |  |  | 856 | -0.721 | -0.4948 | Yes |
| 51 | ANLN |  |  | 860 | -0.733 | -0.4851 | Yes |
| 52 | FEN1 |  |  | 862 | -0.736 | -0.4729 | Yes |
| 53 | HMMR |  |  | 863 | -0.740 | -0.4595 | Yes |
| 54 | CCNB2 |  |  | 865 | -0.745 | -0.4472 | Yes |
| 55 | RRM1 |  |  | 866 | -0.749 | -0.4337 | Yes |
| 56 | NDC80 |  |  | 869 | -0.762 | -0.4222 | Yes |
| 57 | KIF23 |  |  | 873 | -0.772 | -0.4117 | Yes |
| 58 | TMPO |  |  | 875 | -0.778 | -0.3988 | Yes |
| 59 | KIF20B |  |  | 876 | -0.779 | -0.3847 | Yes |
| 60 | CKAP2 |  |  | 878 | -0.781 | -0.3718 | Yes |
| 61 | RAD51AP1 |  |  | 894 | -0.853 | -0.3737 | Yes |
| 62 | CENPE |  |  | 899 | -0.873 | -0.3626 | Yes |
| 63 | SMC4 |  |  | 902 | -0.886 | -0.3488 | Yes |
| 64 | CCNE2 |  |  | 907 | -0.895 | -0.3373 | Yes |
| 65 | CKS1B |  |  | 909 | -0.912 | -0.3220 | Yes |
| 66 | HJURP |  |  | 910 | -0.921 | -0.3053 | Yes |
| 67 | CKS2 |  |  | 911 | -0.922 | -0.2886 | Yes |
| 68 | GTSE1 |  |  | 912 | -0.941 | -0.2716 | Yes |
| 69 | KIF11 |  |  | 913 | -0.957 | -0.2543 | Yes |
| 70 | CLSPN |  |  | 916 | -0.964 | -0.2392 | Yes |
| 71 | TYMS |  |  | 917 | -0.965 | -0.2217 | Yes |
| 72 | DLGAP5 |  |  | 923 | -0.998 | -0.2095 | Yes |
| 73 | UBE2C |  |  | 928 | -1.108 | -0.1941 | Yes |
| 74 | NUSAP1 |  |  | 929 | -1.112 | -0.1740 | Yes |
| 75 | TPX2 |  |  | 930 | -1.124 | -0.1537 | Yes |
| 76 | ATAD2 |  |  | 933 | -1.162 | -0.1350 | Yes |
| 77 | CENPF |  |  | 935 | -1.200 | -0.1144 | Yes |
| 78 | HMGB2 |  |  | 937 | -1.238 | -0.0932 | Yes |
| 79 | CDK1 |  |  | 938 | -1.248 | -0.0707 | Yes |
| 80 | RRM2 |  |  | 940 | -1.403 | -0.0464 | Yes |
| 81 | MKI67 |  |  | 941 | -1.407 | -0.0210 | Yes |
| 82 | TOP2A |  |  | 942 | -1.417 | 0.0046 | Yes |
Table: GSEA details [plain text format]

  

Fig 2: SEURAT\_CELLCYCLE: Random ES distribution      
 Gene set null distribution of ES for **SEURAT\_CELLCYCLE**
